# Optimizing water and phosphorus management to improve hay yield and water‐ and phosphorus‐use efficiency in alfalfa under drip irrigation

**DOI:** 10.1002/fsn3.1530

**Published:** 2020-03-19

**Authors:** Qianbing Zhang, Junying Liu, Xuanshuai Liu, Shengyi Li, Yanliang Sun, Weihua Lu, Chunhui Ma

**Affiliations:** ^1^ The College of Animal Science & Technology Shihezi University Shihezi China

**Keywords:** alfalfa, available phosphorus, drip irrigation, hay yield, total phosphorus, water‐ and phosphorus‐use efficiency

## Abstract

Alfalfa (*Medicago sativa* L.) is an important forage legume in arid areas, but limited water resources and low fertilizer utilization have restricted its agricultural development. Meanwhile, studies on the effects of integrated water and phosphorus on production performance and water‐use efficiency and phosphorus‐use efficiency of alfalfa, especially on hay yield, phosphorus accumulation, and total phosphorus uptake are rarely reported under drip irrigation. The treatments were a factorial combination of three irrigation rates (5,250, 6,000, and 6,750 m^3^/ha per year) and four P rates (0, 50, 100, and 150 kg/ha per year) and consisted of 12 treatments for water and P management, arranged in a randomized complete block design with three replicates. Total hay yield and water‐use efficiency and phosphorus‐use efficiency of alfalfa in P_2_ treatment were significantly greater than those in the P_1_ and P_3_ treatments (*p* < .05), and the total hay yield of alfalfa with phosphorus application increased by 7.43%–29.87% compared with that in the nonphosphorus (P_0_) treatment under the same irrigation amount. The total phosphorus and available phosphorus concentrations in the 0–20 cm soil layer were greater than those in the 20–40 cm and 40–60 cm soil layers compared with those in the P_0_ treatment. Correlation analyses showed that total hay yield was significantly positively correlated with total phosphorus uptake and water‐use efficiency (*p* < .01). The accumulated phosphorus concentration was significantly positively correlated with total phosphorus and available phosphorus concentration (*p* < .01) and was positively correlated with the phosphorus‐use efficiency (*p* < .05). The membership function method was used to evaluate all the indicators, and the three treatments that had the greatest influence on the production performance of alfalfa were, in order, W_2_P_2_ > W_3_P_2_ > W_1_P_2_. Therefore, an irrigation rate of 6,000 m^3^/ha and a phosphorus application rate of 100 kg/ha per year should be considered as the best management for both high yield and water‐use efficiency and phosphorus‐use efficiency of alfalfa.

## Graphical abstract

Irrigation amounts were 6,000 m^3^/ha and phosphorus application was 100 kg/ha, which was the optimal water–phosphorus combination model for alfalfa to produce high quality and high yield under drip irrigation.

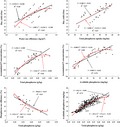

## 1 INTRODUCTION

Alfalfa (*Medicago sativa* L.) is a perennial leguminous forage that has the largest area of forage cultivation in China because of its high yield, rich protein concentration, strong biological nitrogen fixation ability, and wide adaptability (Huang et al., 2018). However, alfalfa consumes large amounts of water; water has been the major factor limiting its development, especially in dry areas with little precipitation (Gu et al., 2018), and irrigation is required to maximize alfalfa yields (Wang et al., 2018). Therefore, water‐saving irrigation for alfalfa cultivation has become the fundamental way to sustainably develop the alfalfa industry in Xinjiang. Alfalfa has many branches and abundant stems and leaves, so it needs high levels of nutrients for growth and development. Phosphorus is a basic plant nutrient constitutes nucleic acids, phospholipids, adenosine triphosphates, and many coenzymes. It is involved in the synthesis of substances and various physiological and biochemical processes in plants (Syers, Johnston, & Curtin, 2008). However, the gray desert soil in Xinjiang is a typical phosphorus‐deficient soil with a very low available phosphorus concentration, which limits the development of agriculture in Xinjiang. Initially, the application of phosphate fertilizer was to alleviate this situation; however, the solubility of phosphorus is low, its mobility is poor, and it is easily fixed by metal ions after the application of phosphate fertilizer to the soil, making the phosphorus‐use efficiency in a growing season only 5%–25% (Yang, Wang, et al., 2012). All the rest of the phosphorus is in the form of recalcitrant phosphorus residues in soil, which enriches the phosphorus in the soil. This situation restricts the growth and development of plants while causing phosphorus pollution in the soil and wasting limited phosphorus resources (Mai, Xue, Feng, Yang, & Tian, 2018). However, without the addition of phosphorus fertilizer over a long period, the soil available phosphorus concentration will gradually decrease (Yao et al., 2012), and the crop yield will decrease accordingly. In the past few decades, crop yields have not increased proportionally with increasing fertilizer inputs (Wang et al., 2017). Therefore, it is necessary to improve fertilizer‐use efficiency through appropriate fertilization to ensure high crop yield and maintain soil fertility (Song et al., 2018).

Some studies have demonstrated that phosphorus and water have synergistic effects on plant growth (Thompson, Doerge, & Godin, 2000). Within a certain range of water and phosphorus levels, irrigation can effectively improve the absorption, transformation, and utilization of fertilizers by crops. Appropriate fertilization can reduce the negative effects of soil water deficiency on crop growth and development to a certain extent (Yang, Guo, Wang, Yang, & Yang, 2012) and can also increase the phosphorus concentration in plants (Gu et al., 2018) with increased phosphorus uptake. Therefore, the appropriate management of water and fertilizer can not only increase crop yield and reduce irrigation and phosphorus application but can also reduce total phosphorus and increase available phosphorus in soil (Schärer et al., 2010). Meanwhile, the combination of water and fertilizer can effectively improve the water‐use efficiency and phosphorus‐use efficiency of alfalfa (Lenssen et al., 2010), which is beneficial for reducing the loss of agricultural water in the field and the excessive use of phosphate fertilizers. In many studies on water and fertilizer management in alfalfa, single factors such as water and phosphorus have often been used to evaluate the influence of different irrigation amounts or phosphorus application rates on the production performance of alfalfa (Mallarino & Rueber, 2013). However, the relationships between water‐use efficiency and phosphorus‐use efficiency and hay yield, total phosphorus uptake and hay yield, and soil total phosphorus and available phosphorus under both irrigation and phosphorus application are rarely reported. Therefore, this study carried out a 3‐year study on the effects of different irrigation amounts applied via subsurface drip irrigation and phosphorus application rates on hay yield and soil phosphorus concentrations. The objectives of the present study were to (a) examine the effects of different levels of irrigation and phosphorus on yield and water‐use efficiency and phosphorus‐use efficiency in alfalfa and (b) examine the impacts of the phosphorus application on phosphorus concentration accumulation in the plants and the soil. The results of present investigation are of significant importance for providing practical guidance for the development of a reasonable and efficient water and fertilizer irrigation system for high quality and high yield in alfalfa under drip irrigation.

## 2 MATERIALS AND METHODS

### 2.1 Site description

The field experiment was conducted during 2016–2018 at Tianye Group Agricultural Demonstration Park (44°26′N, 85°95′E), Shihezi City, Xinjiang, China. The experimental site was located in a temperate continental climate zone. The area is dry and rainless, and the diurnal temperature varies greatly. The mean annual temperature is 11.2–13.98°C, the annual precipitation was 203.1–394.9 mm, and the annual pan evaporation is approximately 1,000–1,500 mm. The soil type was a gray desert soil (Chinese soil classification) or Aridisol (United States Department of Agriculture (USDA) classification). The previous crop was cotton (*Gossypium* spp.). The physical and chemical properties of the 0–60 cm plow layer soil are shown in Table 1.

**TABLE 1:** Soil indicators of 0–60 cm at experiment station in 2016–2018

Depth (cm)	Years	Organic matter (g/kg)	Alkali‐hydrolyzed nitrogen (mg/kg)	Total nitrogen (g/kg)	Available phosphorus (mg/kg)	Total Phosphorus (g/kg)	Available potassium (mg/kg)	Field capacity (%)	Soil moisture concentration (%)	Soil bulk density (g/cm^3^)	pH value
0–20	2016	25.5	60.8	1.76	25.5	0.23	330.2	24.9	30.1	1.56	7.63
	2017	25.3	72.6	1.61	16.3	0.21	139.6	24.6	29.2	1.48	7.75
	2018	24.9	68.3	1.53	15.7	0.22	132.6	24.2	28.4	1.58	7.83
20–40	2016	23.9	59.4	1.69	11.9	0.20	278.4	24.5	29.8	1.60	7.61
	2017	22.5	68.9	1.55	10.0	0.21	109.7	24.2	28.6	1.52	7.72
	2018	22.1	65.4	1.50	11.7	0.20	99.5	23.9	27.9	1.63	7.8
40–60	2016	20.7	55.4	1.66	7.7	0.17	258.4	24.2	28.5	1.61	7.59
	2017	19.3	67.0	1.52	7.6	0.18	91.7	24.0	27.8	1.54	7.70
	2018	18.7	62.6	1.49	9.6	0.19	87.4	23.7	26.7	1.65	7.78

### 2.2 Experimental design

The experiment was conducted in a split plot based on a randomized complete block design with three replications and consisted of three levels of irrigation rates and four levels of phosphorus fertilization. The three levels of irrigation rates were 5,250 m^3^/ha (W_1_), 6,000 m^3^/ha (W_2_, actual irrigation amounts on high yield of alfalfa in local field), and 6,750 m^3^/ha (W_3_). The four phosphorus fertilizer rates in the experiment were (as P_2_O_5_ equivalent) 0 kg/ha (P_0_), 50 kg/ha (P_1_), 100 kg/ha (P_2_), and 150 kg/ha (P_3_). Three replications were performed for the 12 water–phosphorus management treatments, and each plot size was 5 by 8 m equal 40 m^2^. The distance between each plot is 1.5 m to avoid the lateral movement of water from irrigation level to another.

**FIGURE 1:**
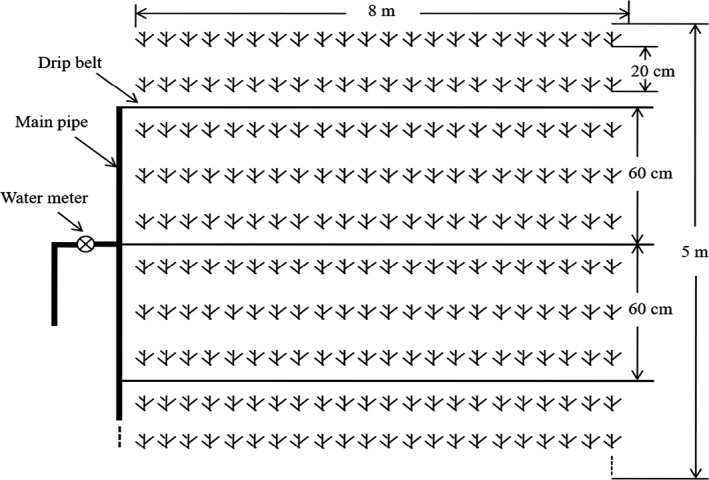
Experimental plot of drip irrigation for alfalfa and layout of drip irrigation tape

The WL354HQ alfalfa seeds (Beijing Zhengdao Ecological Technology Co., Ltd.) were sown on April 19, 2015. The crop was sown with a seed drill at a seed rate of 18 kg/ha with a row spacing of 20 cm, and the sowing depth was 2.0 cm. The full quota of irrigation water per plot was delivered across 8 inlaid drip irrigation belts with working pressure of 0.1 MPa and diameter of 12.5 mm. The dripper spacing of drip irrigation belt was 20 cm, and the irrigation water discharge of drip irrigation belt was 3.2 L/h. The drip irrigation belts were buried under the surface 8–10 cm deep with a spacing of 60 cm. The drip irrigation belt was laid with 3 rows of alfalfa for one pipe (Figure 1). The specific amount of irrigation was controlled by a water meter at the intake of the plot. The main pipe diameter in the drip irrigation system was 75 mm. There were 8 irrigation events during the growth year. The amounts of irrigation water application (IWA) to each plot during the irrigation regime were determined by using the following equation (El‐Mageed, El‐Sherif, Ali, & El‐Wahed, 2017):

$$\text{IWA}=\left(A\times \text{ETc}\times \text{Li}\right)/\left(\text{Ea}\times 1000\right).$$

where IWA is the irrigation water application (m^3^), *A* is the area (m^2^), ETc is the reference evapotranspiration (mm/day), Li is the irrigation intervals (day), and Ea is the application efficiency (%).

**FIGURE 2:**
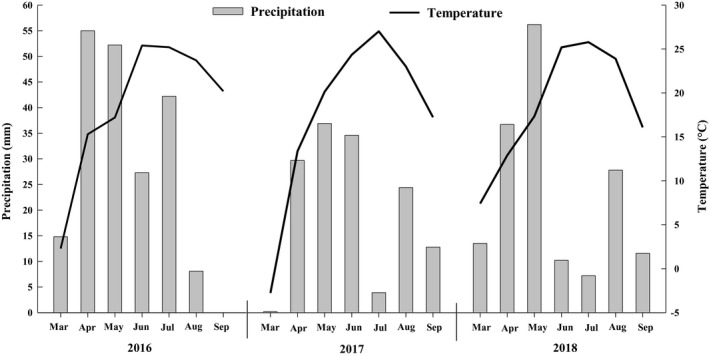
Mean monthly precipitation and temperature during the alfalfa growing seasons in 2016–2018 at the Shihezi meteorological station in Xinjiang

The phosphorus fertilizer was applied together with the irrigation water under drip irrigation, beginning at the branching stage of spring growth following winter dormancy and subsequently 3–5 days after the first, second, and third cuts. The phosphate fertilizer used was monoammonium phosphate (P_2_O_5_ 52%) with good water solubility. To keep the test only affected by the phosphate fertilizer, and based on the monoammonium phosphate (NH_4_H_2_PO_4_) containing nitrogen fertilizer, the effect of nitrogen fertilizer on the production of alfalfa was offset by adding urea (CN_2_H_4_O) to maintain the consistency of the test, as shown in Table 2. The monthly precipitation and average temperatures during the growing seasons in 2016–2018 are presented in Figure 2.

**TABLE 2:** Irrigation amounts and fertilizer application rate per year

Treatments	Irrigation amounts (m^3^/ha)	NH_4_H_2_PO_4_ (kg/ha)	NH_4_H_2_PO_4_ (P_2_O_5_ 52%) (kg/ha)	NH_4_H_2_PO_4_ (N 12.2%) (kg/ha)	CN_2_H_4_O (N 46%) (kg/ha)
W_1_P_0_	5,250	0	0	0	0
W_1_P_1_	5,250	96	50	11.7	51
W_1_P_2_	5,250	192	100	23.4	25.5
W_1_P_3_	5,250	288	150	35.1	0
W_2_P_0_	6,000	0	0	0	0
W_2_P_1_	6,000	96	50	11.7	51
W_2_P_2_	6,000	192	100	23.4	25.5
W_2_P_3_	6,000	288	150	35.1	0
W_3_P_0_	6,750	0	0	0	0
W_3_P_1_	6,750	96	50	11.7	51
W_3_P_2_	6,750	192	100	23.4	25.5
W_3_P_3_	6,750	288	150	35.1	0

### 2.3 Sampling and measurements

#### 2.3.1 Forage yield

The yield of alfalfa was measured by taking a sample of 1 m × 1 m at the early flowering stage (10% blooming) and cutting four times a year. The alfalfa plants in the sample plot (cut to 5 cm) were cut with scissors and weighed, and the yield of fresh alfalfa forage was recorded three times for every treatment. Three samples of 300 g fresh alfalfa were taken back to the laboratory. The samples were first oven‐dried at 105°C for 30 min and then at 65°C to a constant mass. The forage yield (t/ha) was calculated using the following equation:

$$\text{Hay yield}=\text{FY}\times \left(1-\text{MC}\right)$$

where FY is the fresh yield (t/ha) and MC is the moisture concentration.

In the process of measuring alfalfa hay yield, three fresh alfalfa samples were dried and crushed. The phosphorus concentration was determined using the molybdenum‐antimony antispectrophotometric method (Fan, Du, et al., 2016). The total phosphorus uptake of alfalfa was the sum of the phosphorus concentration in the four cuttings of alfalfa plants. The total phosphorus uptake of alfalfa was calculated using the following equation:

Total phosphorus uptake=THY×APC

where THY was total hay yield (kg/ha) and APC is the accumulated phosphorus concentration.

#### 2.3.2 Phosphorus‐use efficiency

The phosphorus‐use efficiency of alfalfa was calculated using the following equation:

$$\text{PUE}=\left(\text{Yi}-\text{Yc}\right)/\text{TPA}$$

where PUE was phosphorus‐use efficiency (%), Yi was total phosphorus uptake in the phosphorus application treatment, Yc is the total phosphorus uptake in the Nonphosphorus (P_0_) treatment, and TPA is the total phosphorus application in the phosphorus application treatment.

#### 2.3.3 Water‐use efficiency

The hay yield water‐use efficiency of alfalfa was calculated using the following equation (El‐Mageed et al., 2017):

WUE=THY/WA

where WUE is the hay yield water‐use efficiency (kg/m), THY is the total hay yield (kg/ha), and WA is the water applied (m^3^/ha).

#### 2.3.4 Soil phosphorus concentration

The “S”‐shaped sampling method was adopted to take soil samples at 0–20 cm, 20–40 cm, and 40–60 cm with the soil drill in each plot. Samples of different soil depths were taken at one site after taking the fourth cut, with five replications. Impurities such as roots and stones were removed, and the soil samples were brought back to the laboratory to dry in a cool and ventilated place. The soil was sieved through a 2 mm sieve and placed in a plastic self‐sealing bag for the determination of total phosphorus and available phosphorus in soil. Total phosphorus was determined by the sulfuric acid–perchloric acid decoction molybdenum antimony colorimetric method, and available phosphorus was determined by the NaHCO_3_ extraction molybdenum antimony colorimetric method (Mehlich, 1984).

### 2.4 Statistical analysis

All the plant data collected were statistically analyzed by SPSS 20.0 (Statistical Product and Service Solutions, USA) using analysis of variance, and testing of the obtained results was performed by Fisher's least significant difference (Duncan's) test with significance determined at the 5% level. Linear and nonlinear regression analysis methods were used to identify the relationships between the indicators. The figures were prepared with Origin 8.0 (OriginLab OriginPro, USA). The membership function evaluation method was used to comprehensively evaluate the optimum treatment, and the specific formula was as follows:

$$\text{UX}\;\left(+\right)=\left(\text{Xij}-\text{Ximin}\right)/\left(\text{Ximax}-\text{Ximin}\right).$$

$$\text{UX}\;\left(-\right)=1-\text{UX}\;\left(+\right).$$

where X is the measured value of each index of the sample, UX (+) is the positive correlation membership function value of each index, and UX (−) is the negative correlation membership function value of each index.

Pearson's correlation analysis was used to analyze the degrees of correlation among the alfalfa variables. Pearson's correlation coefficient is a numerical value between 1 and −1, where 1 means that the variables are completely positively correlated, 0 means that the variables are not correlated, and −1 means that *t* the variables are completely negatively correlated.

## 3 RESULTS

### 3.1 Total hay yield of alfalfa

The total hay yield first increased and then decreased with increasing phosphorus application and reached a maximum in the P_2_ treatment at the same irrigation level. The hay yield in the P_2_ treatment was significantly higher than that in the P_1_ and P_3_ treatments (*p* < .05) (Table 3). The total hay yield in W_2_ and W_3_ treatments were significantly higher than that in the W_1_ treatment (*p* < .05), except that the hay yield in the W_3_ treatment was significantly higher than that in the W_1_ and W_2_ treatments under the P_0_, P_1_, and P_3_ treatments in 2018 (*p* < .05). The total hay yield in the phosphorus application treatments increased by 7.43%–29.87% compared with nonphosphorus (P_0_) treatment under the same irrigation amounts, and the stimulation effect was obvious. The total hay yield reached a maximum under W_2_P_2_ treatment, at 23.16, 22.97, and 20.55 t/ha in 2016–2018, respectively, and the hay yield decreased by 0.82% and 13.64% in 2017 and 2018, respectively, compared with that in 2016. This indicated that the total hay yield decreased gradually with time.

**TABLE 3:** Total hay yield of alfalfa under different water and phosphorus conditions (t/ha)

Treatments	2016	2017	2018
W_1_P_0_	19.21 ± 0.19Bc	18.93 ± 0.18Bc	14.93 ± 0.55Bc
W_1_P_1_	20.84 ± 0.22Cb	20.66 ± 0.01Bb	17.21 ± 0.11Cb
W_1_P_2_	21.76 ± 0.22Ca	22.43 ± 0.02Ba	18.96 ± 0.03Ba
W_1_P_3_	21.19 ± 0.26Bb	20.92 ± 0.09Bb	17.54 ± 0.46Cb
W_2_P_0_	20.44 ± 0.22Ac	19.47 ± 0.04Ad	15.83 ± 0.40Bc
W_2_P_1_	22.18 ± 0.17Ab	21.09 ± 0.04Ac	18.18 ± 0.54Bb
W_2_P_2_	23.16 ± 0.35Aa	22.97 ± 0.08Aa	20.55 ± 0.16Aa
W_2_P_3_	22.26 ± 0.22Ab	21.74 ± 0.04Ab	18.86 ± 0.08Bb
W_3_P_0_	20.08 ± 0.24Ac	19.64 ± 0.23Ad	16.22 ± 0.40Ad
W_3_P_1_	21.57 ± 0.13Bb	21.17 ± 0.03Ac	18.95 ± 0.14Ac
W_3_P_2_	22.60 ± 0.25Ba	22.97 ± 0.09Aa	20.30 ± 0.20Aa
W_3_P_3_	21.56 ± 0.37Ab	21.60 ± 0.03Ab	19.48 ± 0.21Ab

Note

Different capital letters within the same column mean significant difference at the .05 level, different small letters within the same column mean significant difference at .05 level.

### 3.2 Accumulated phosphorus concentration and total phosphorus uptake of alfalfa

The accumulated phosphorus concentration and total phosphorus uptake of alfalfa increased gradually in 2016 and increased first and then decreased in 2017 and 2018 with the increase in phosphorus application at the same irrigation level (Table 4). The accumulated phosphorus concentration and total phosphorus uptake in the P_1_, P_2_, and P_3_ treatments were significantly higher than those in the P_0_ treatment. The accumulated phosphorus concentration in the phosphorus treatments increased by 19.99%–36.87% compared with that in the P_0_ treatment (*p* < .05). The total phosphorus uptake in phosphorus treatments increased by 29.23%–60.16% compared with that in the P_0_ treatment (*p* < .05). The total phosphorus uptake in the W_2_ and W_3_ treatments increased by 2.06%–13.81% and 0.73%–13.14%, respectively, compared with that in the W_1_ treatment (*p* < .05). This indicated that the addition of phosphate fertilizer effectively promoted the absorption of phosphorus in plants.

**TABLE 4:** Accumulated phosphorus concentration and total phosphorus uptake of alfalfa under different treatments

Treatments	Accumulated phosphorus concentration (%)			Total phosphorus uptake (kg/ha)		
	2016	2017	2018	2016	2017	2018
W_1_P_0_	0.7276 ± 0.0064Bc	0.7432 ± 0.0032Cd	0.8289 ± 0.0057Ab	35.78 ± 0.52Bd	35.20 ± 0.48Bd	30.93 ± 1.45Bd
W_1_P_1_	0.9330 ± 0.0224Ab	0.9049 ± 0.0019Cc	0.9946 ± 0.0115ABa	49.44 ± 1.31Bc	46.74 ± 0.08Cc	42.27 ± 0.1Cc
W_1_P_2_	0.9575 ± 0.0197Bb	0.9737 ± 0.0013Ba	1.0147 ± 0.0020Ba	52.76 ± 0.98Cb	54.85 ± 0.05Ca	47.44 ± 0.06Ba
W_1_P_3_	1.0234 ± 0.0049Ba	0.9540 ± 0.0016Cb	1.0117 ± 0.0136Ba	54.85 ± 0.37Ba	49.99 ± 0.25Cb	44.08 ± 0.56Cb
W_2_P_0_	0.7245 ± 0.0009Bd	0.7661 ± 0.0028Ad	0.8291 ± 0.0148Ad	37.99 ± 0.45Ad	37.23 ± 0.18Ad	32.80 ± 1.44ABd
W_2_P_1_	0.8985 ± 0.0107Bc	0.9207 ± 0.0018Ac	1.0125 ± 0.0188Ac	50.46 ± 0.53ABc	48.58 ± 0.18Ac	45.46 ± 0.54Bc
W_2_P_2_	0.9379 ± 0.0049Bb	1.0332 ± 0.0033Aa	1.0510 ± 0.0001Aa	54.96 ± 0.62Bb	59.49 ± 0.38Aa	53.99 ± 0.53Aa
W_2_P_3_	1.0441 ± 0.0006Aa	1.0130 ± 0.0023Ab	1.0401 ± 0.0081Ab	58.19 ± 0.54Aa	55.34 ± 0.21Ab	48.67 ± 0.15Bb
W_3_P_0_	0.7471 ± 0.0025Ad	0.7550 ± 0.0027Bc	0.8220 ± 0.0100Ad	38.32 ± 0.21Ad	37.15 ± 0.32Ad	33.36 ± 1.21Ad
W_3_P_1_	0.9433 ± 0.0095Ac	0.9077 ± 0.0001Bb	0.9886 ± 0.0041Bc	51.16 ± 0.68Ac	48.01 ± 0.07Bc	46.84 ± 0.16Ac
W_3_P_2_	1.0169 ± 0.0107Ab	0.9594 ± 0.0037Ca	1.0502 ± 0.0058Aa	57.88 ± 0.40Aa	55.25 ± 0.4Ba	53.43 ± 0.47Aa
W_3_P_3_	1.0457 ± 0.0058Aa	0.9645 ± 0.0021Ba	1.0150 ± 0.0010Bb	56.49 ± 0.25Bb	52.05 ± 0.05Bb	49.87 ± 0.57Ab

Note

Different capital letters within the same column mean significant difference at the .05 level, different small letters within the same column mean significant difference at .05 level.

### 3.3 Water‐use efficiency and phosphorus‐use efficiency of alfalfa

The water‐use efficiency of alfalfa increased first and then decreased with increasing phosphorus application and reached a maximum in the P_2_ treatment at the same irrigation level. The water‐use efficiency of the P_2_ treatment was significantly higher than that in the P_1_, P_3_, and P_0_ treatments (*p* < .05), and the P_2_ treatment increased by 12.55%–29.79% compared with that in the P_0_ treatment (Table 5). It was indicated that phosphorus application significantly improved the water‐use efficiency. The water‐use efficiency in the W_1_ treatment was significantly higher than that in the W_2_ and W_3_ treatments (*p* < .05). Thus, the water‐use efficiency decreased with the increasing irrigation amount. The phosphorus‐use efficiency of alfalfa decreased with an increasing phosphorus application at the same irrigation level.

**TABLE 5:** Water‐ and phosphorus‐use efficiency of alfalfa under different treatments

Treatments	Water‐use efficiency (kg/m^3^)			Phosphorus‐use efficiency (%)		
	2016	2017	2018	2016	2017	2018
W_1_P_0_	3.66 ± 0.05Ac	3.60 ± 0.03Ac	2.84 ± 0.04Ac	—	—	—
W_1_P_1_	3.97 ± 0.06Ab	3.93 ± 0.01Ab	3.28 ± 0.02Ab	27.32 ± 3.64Aa	23.06 ± 1.12Aa	22.68 ± 3.09Aa
W_1_P_2_	4.14 ± 0.06Aa	4.27 ± 0.03Aa	3.61 ± 0.03Aa	16.99 ± 1.49Bb	19.64 ± 0.53Bb	16.52 ± 1.50Ab
W_1_P_3_	4.04 ± 0.07Ab	3.98 ± 0.02Ab	3.34 ± 0.09Ab	12.72 ± 0.59Bc	9.86 ± 0.16Bc	8.76 ± 1.34Bc
W_2_P_0_	3.41 ± 0.05Bc	3.24 ± 0.01Bc	2.64 ± 0.07Bc	—	—	—
W_2_P_1_	3.70 ± 0.04Bb	3.52 ± 0.01Bb	3.03 ± 0.09Bb	24.93 ± 1.95Aa	22.71 ± 0.01ABa	25.31 ± 1.81Aa
W_2_P_2_	3.86 ± 0.08Ba	3.83 ± 0.04Ba	3.42 ± 0.03Ba	16.97 ± 0.17Bb	22.26 ± 0.56Ab	21.19 ± 1.98Ab
W_2_P_3_	3.71 ± 0.05Bb	3.62 ± 0.02Bb	3.14 ± 0.02Bb	13.47 ± 0.06Ac	12.07 ± 0.02Ac	10.58 ± 1.07ABc
W_3_P_0_	2.97 ± 0.05Cc	2.91 ± 0.03Cc	2.40 ± 0.06Cc	—	—	—
W_3_P_1_	3.20 ± 0.03Cb	3.14 ± 0.04Cb	2.81 ± 0.02Cb	25.68 ± 1.78Aa	21.73 ± 0.51Ba	26.96 ± 2.74Aa
W_3_P_2_	3.35 ± 0.05Ca	3.40 ± 0.02Ca	3.01 ± 0.03Ca	19.55 ± 0.61Ab	18.10 ± 0.73Cb	20.07 ± 1.68Ab
W_3_P_3_	3.19 ± 0.08Cb	3.20 ± 0.05Cb	2.89 ± 0.03Cb	12.11 ± 0.03Bc	9.93 ± 0.25Bc	11.01 ± 1.18Ac

Note

Different capital letters within the same column mean significant difference at the .05 level, different small letters within the same column mean significant difference at .05 level.

### 3.4 Soil total phosphorus

**FIGURE 3:**
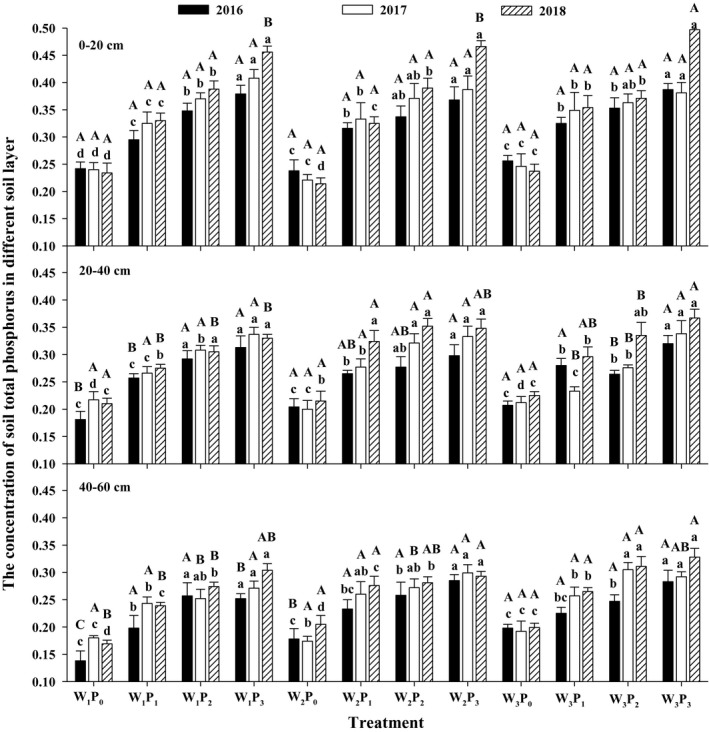
Soil total phosphorus concentration under different treatments (g/kg). Different capital letters indicate significant differences in the different irrigation levels under the same P application conditions (*p* < .05). Different small letters indicate significant differences in the different P levels under the same irrigation conditions (*p* < .05)

The total phosphorus concentration of the soil in the phosphorus treatment increased gradually with the increase in the phosphorus application rate and reached a maximum in the P_3_ treatment, except in the W_1_ treatment in the 40–60 cm soil layer in 2016 (Figure 3). The total phosphorus concentration in P_3_ treatment was significantly higher than that in the P_0_ treatment at 0–60 cm at the same irrigation level (*p* < .05). The total phosphorus in the phosphorus treatment increased by 21.9%–117.8% in the 0–20 cm soil layer, by 9.9%–72.9% in the 20–40 cm soil layer, and by 13.60%–82.6% in the 40–60 cm soil layer compared to those in the P_0_ treatment. The total phosphorus in the no‐phosphorus treatment in the 0–20 cm soil layer showed a decreasing trend, but total phosphorus showed an increasing trend in 0–60 cm soil layer under the phosphorus treatment. The total phosphorus concentration decreased gradually with the increasing soil depth, reaching a maximum in the 0–20 cm soil layer and a minimum in the 40–60 cm soil layer.

### 3.5 Soil available phosphorus

**FIGURE 4:**
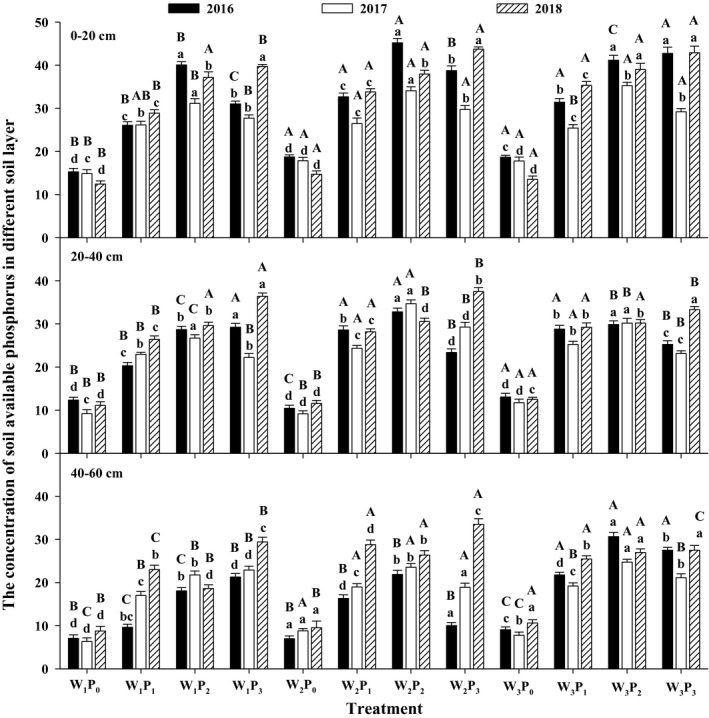
Soil available phosphorus concentration under different treatments (%).Different capital letters indicate significant differences in the different irrigation levels under the same P application conditions (*p* < .05). Different small letters indicate significant differences in the different P levels under the same irrigation conditions (*p* < .05)

The available phosphorus increased gradually or increased first and then decreased with the increase in the phosphorus application rate and reached a maximum in the P_2_/P_3_ treatments, and the available phosphorus in the phosphorus treatment was significantly higher than that in the P_0_ treatment at the same depth (*p* < .05) (Figure 4). The available phosphorus in phosphorus treatment increased by 42.89%–218.97% in the 0–20 cm soil layer, by 64.75%–279.13% in the 20–40 cm soil layer, and by 36.40%–262.34% in the 40–60 cm soil layer compared to that in the P_0_ treatment. The available phosphorus in the W_2_ and W_3_ treatments was significantly higher than that in the W_1_ treatment at the same depth (*p* < .05). The available phosphorus in the P_0_ treatment showed a decreasing trend in the 0–20 cm soil layer. The available phosphorus was mainly concentrated in the 0–20 cm layer and decreased with increasing in soil depth.

### 3.6 Pearson's correlation analysis

The hay yield of alfalfa was significantly positively correlated with total phosphorus uptake and water‐use efficiency (*p* < .01). The accumulated phosphorus concentration was significantly positively correlated with the total phosphorus and available phosphorus concentration (*p* < .01) and was positively correlated with the phosphorus‐use efficiency (*p* < .05). Total phosphorus was significantly positively correlated with available phosphorus (*p* < .01). There were no significant correlations among the other indicators (*p* > .05).

### 3.7 Linear nonlinear equations between significantly related paired indicators

**FIGURE 5:**
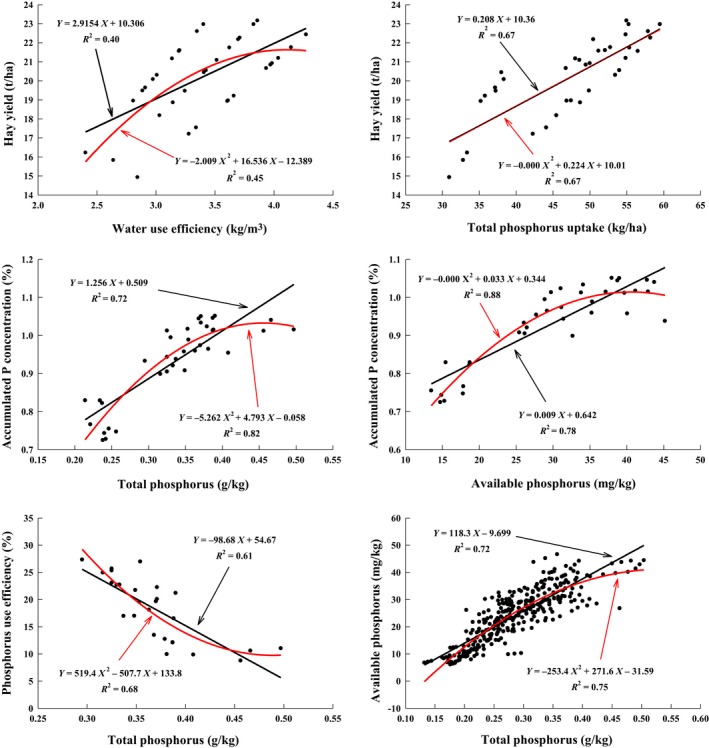
Linear and nonlinear equations between extremely significantly related paired indicators

Figure 5 was obtained by fitting the extremely significantly related paired indicators to the one‐dimensional linear and polynomial equations in Table 6 above. The total phosphorus and available phosphorus were matched with other indicators using only 0–20 cm data; while the total phosphorus and available phosphorus were fitted with all data from 0 to 60 cm, and P_0_ was removed from the phosphorus‐use efficiency fitting data. The determinant coefficient (*R* ^2^) values for the phosphorus accumulation concentration and phosphorus‐use efficiency were small, so they were not included in the figure below. The *R* ^2^ of available phosphorus and phosphorus accumulation was the largest, followed by that of total phosphorus and available phosphorus. This indicated that the accumulation of phosphorus came from the available phosphorus concentration in the soil, while the available phosphorus concentration in the soil was limited by the supply of total phosphorus. Maintaining the total phosphorus concentration in the soil was beneficial for increasing the level of available phosphorus, thus ensuring the supply of phosphorus nutrients in alfalfa.

**TABLE 6:** The correlation analysis of each index of alfalfa under different treatments

Index	Hay yield	Accumulated phosphorus concentration	Total phosphorus uptake	Water‐use efficiency	Phosphorus‐use efficiency	Total phosphorus
Accumulated phosphorus concentration	−0.255					
Total phosphorus uptake	0.837**	0.312				
Water‐use efficiency	0.529**	−0.293	0.316			
Phosphorus‐use efficiency	0.020	−0.400*	−0.200	−0.055		
Total phosphorus	−0.302	0.544**	0.008	−0.211	−0.782**	
Available phosphorus	−0.015	0.576**	0.308	−0.358	−0.379	0.495**

* Significant correlation was found at the .05 level (bilateral).

** Significant correlation was found at the .01 level (bilateral).

### 3.8 Membership function analysis

Since each treatment performed differently for the different indicators, it was not sufficient to evaluate the optimal irrigation amounts and phosphorus application with any single indicator. The total hay yield, accumulated phosphorus concentration, total phosphorus uptake, water‐use efficiency, and phosphorus‐use efficiency were positive indicators, while total phosphorus concentration was a negative indicator. The membership function values of the averages of 7 indicators were sorted by their comprehensive value. The larger the average value, the higher the comprehensive value, and vice versa. The comprehensive assessment of various indicators of alfalfa by membership function analysis showed that W_2_P_2_ > W_3_P_2_ > W_1_P_2_ were the top three treatments for alfalfa production performance (Table 7).

**TABLE 7:** Comprehensive evaluation of each index

Index	W_1_P_1_	W_1_P_2_	W_1_P_3_	W_2_P_1_	W_2_P_2_	W_2_P_3_	W_3_P_1_	W_3_P_2_	W_3_P_3_
Total hay yield	0.0013	0.5569	0.1189	0.3442	1.0000	0.5207	0.3742	0.8974	0.4931
Accumulated phosphorus concentration	0.0446	0.4496	0.6039	0.0418	0.7218	0.1000	0.0700	0.7375	0.7329
Total phosphorus uptake	0.0003	0.5537	0.3493	0.2020	1.0000	0.7920	0.2523	0.9373	0.6657
WUE	0.6508	1.0002	0.7237	0.3852	0.7543	0.4857	0.0125	0.2024	0.0006
PUE	0.9706	0.5073	0.0001	0.9681	0.6765	0.1113	1.0000	0.6139	0.0400
Total phosphorus	1.0000	0.4970	0.0818	0.8970	0.5212	0.1485	0.7333	0.5545	0.0152
Available phosphorus	0.0003	0.7545	0.4802	0.3272	1.0000	0.8611	0.3058	0.9496	0.9333
Average	0.3811	0.6170	0.3369	0.4522	0.8105	0.4313	0.3926	0.6989	0.4115
Rank	8	3	9	4	1	5	7	2	6

## 4 DISCUSSION

### 4.1 Effects of water and phosphorus management on hay yield, phosphorus accumulation, and phosphorus uptake of alfalfa

The irrigation amount and phosphorus application rate had significant effects on the total hay yield of alfalfa. The research demonstrated that phosphorus application could help to increase the chlorophyll concentration in alfalfa leaves, thereby improving the photosynthetic rate, and then increase the phosphorus concentration in alfalfa plants, resulting in higher yields in phosphorus‐deficient soil (Song et al., 2018). In this study, phosphorus application significantly increased the hay yield, and the accumulated phosphorus concentration and the phosphorus uptake of alfalfa increased gradually with increasing phosphorus application (Tables 3 and 4). Therefore, phosphorus application within a certain range promoted biomass accumulation in alfalfa, promoted phosphorus accumulation in alfalfa plants and increasing the hay yield. However, excessive phosphorus application resulted in a decrease in dry matter quality. There is a certain threshold for the phosphorus absorption by alfalfa plants. Below this threshold, P can promote alfalfa growth and development (Bai et al., 2013). When the phosphorus application exceeded the maximum absorption of phosphorus by alfalfa, the hay yield of the alfalfa plants decreased, and phosphorus had a negative impact on plant growth and development. In this study, with the increase in phosphorus application, the accumulated phosphorus concentration and the total phosphorus uptake increased gradually in 2016 (Table 4), while the hay yield increased first and then decreased (Table 3). Therefore, phosphorus application can improve alfalfa growth. However, excessive phosphorus uptake by alfalfa could have a competitive effect on the uptake mechanisms for other nutrients, resulting in unbalanced nutrition, which would reduce plant yield (Shabani et al., 2011).

As an indispensable substance for crop growth, water not only promotes nutrient uptake and transport, but also directly affects a series of metabolic reactions in crops (Mahfouz, Megawer, & Maher, 2020; Zhang, Liu, Yu, Lu, & Ma, 2020). The total hay yield of alfalfa in the W_2_ and W_3_ treatments was significantly higher than that in the W_1_ treatment under the same phosphorus application (Table 3). This result indicates that the increase in irrigation amounts was more conducive to the improvement of hay yield than the increase in phosphorus application. Studies have shown that irrigation can increase the growth rate and leaf area of alfalfa (Avramova et al., 2015), enhance the photosynthetic leaves of alfalfa and increase the accumulation of photosynthetic products, thereby improving the hay yield (Li, Wan, Wang, & Li, 2018). Low soil moisture levels lead to crop water deficits and inhibit crop growth (Jia et al., 2009). Therefore, the effect of water stress on photosynthesis is reflected in the change of plant biomass. In this study, phosphorus application improved the growth of alfalfa and then increases the hay yield under drought stress (Table 3). Studies have shown that phosphorus regulates root growth under soil water deficit conditions mainly by changing the water status of roots, improving the root water potential, and increasing the absorption of soil water by the root system and the amount of transpiration and evaporation, and then promoting the growth of aboveground and underground plant parts (Liu et al., 2015). In addition, phosphorus application can significantly enhance the stability of tissue and cell membranes, increase stomatal conductance and reduce sensitivity to drought, thereby enhancing drought resistance and improving alfalfa growth (Mickky, Abbas, & El‐Shhaby, 2018).

### 4.2 Effects of water and phosphorus management on water‐use efficiency and phosphorus‐use efficiency of alfalfa

The water‐use efficiency and phosphorus‐use efficiency are important criteria for determining whether the irrigation and phosphorus application amounts are reasonable. The water‐use efficiency is a physiological index used to describe the growth of alfalfa, especially the relationship between harvest yield and crop water consumption (Lamm, Harmoney, Aboukheira, & Johnson, 2012). In this study, the water‐use efficiency decreased with increasing irrigation amounts, and increased first and then decreased with increasing phosphorus application (Table 5). This result indicated that fertilization had obvious water regulating effect, and proper fertilization could improve water‐use efficiency (Thompson et al., 2000). Fertilization can increase the soil water holding capacity (Wang, Liu, & Dang, 2011), and successfully matching fertilizer availability with crop absorption improves water‐use efficiency and increases yield (Agami, Alamri, El‐Mageed, Abousekken, & Hashem, 2018). Other studies have indicated that fertilizers increase the formation of soil aggregates (>0.25 mm) and the levels of nutrients in soil (Liu et al., 2013). Most importantly, the application of phosphate fertilizer promotes plant root development, improves water absorption capacity in roots, and thus improves water‐use efficiency (Fang, Xu, Turner, & Li, 2010). Phosphorus application is also beneficial to the distribution of photosynthetic products in the aboveground parts of the plant, which is very important for improving yield and water‐use efficiency (Hu et al., 2015).

Research has shown that phosphorus application can promote water‐use efficiency, but additional water does not promote phosphorus‐use efficiency. Phosphorus‐use efficiency is related to the degree to which plants mobilize phosphorus from insoluble sources or absorb soluble phosphorus from the soil solutions (Shenoy & Kalagudi, 2005). Elevated mineral nutrient availability occurs when the soil undergoes drying and rewetting cycles under lower irrigation levels. More intense drying (such as that occurring at high temperatures or over long durations) leads to increased mineralization when the soil is re‐watered compared with that in continuously wet soil (Bünemann et al., 2013). Phosphorus had high solubility in W_3_, and water can act as a solvent to dissolve less‐soluble phosphorus; the dissolution reduces total phosphorus and increases available phosphorus (Williams, King, Duncan, Pease, & Penn, 2018). Under suitable irrigation conditions, alfalfa phosphorus uptake and phosphorus‐use efficiency could be improved (Tables 4 and 5). In this study, the phosphorus‐use efficiency ranged from 8.76% to 26.96%, which was improved compared with the average phosphorus‐use efficiency of 5%–25%. This result indicated that optimizing water–phosphorus management could improve the phosphorus‐use efficiency of alfalfa under drip irrigation.

### 4.3 Effects of water and phosphorus management on soil total phosphorus and available phosphorus

The phosphorus application and irrigation amounts had significant influence on the changes in total phosphorus and available phosphorus concentration in the different soil layers. The results showed that the soil total phosphorus and available phosphorus were the highest in the 0–20 cm soil layer and decreased gradually with increasing soil depth, and total phosphorus and available phosphorus in the 0–20 cm soil layer were significantly greater than that in the 20–60 cm (Figures 3 and 4). This is mainly because the total phosphorus and available phosphorus in the soil accumulated at 0–20 cm, while the soil layer at 20–60 cm showed a slightly increasing deficit. The application of phosphate fertilizer to the soil accelerates the recycling of organic matter in the soil, which reduce the adsorption of phosphorus in the soil, promotes the dissociation of phosphorus in the soil, and improves the phosphorus fertility of the soil (Baker, Johnson, Confesor, & Crumrine, 2017). Meanwhile, phosphate fertilizer enters the soil with water and tends to accumulate on the surface; the root system in the soil brings deeper soil nutrients to the surface to provide nutrients for the aboveground plant parts (Fan, Mcconkey, Wang, & Janzen, 2016), thereby increasing the total phosphorus and available phosphorus concentration in the 0–20 cm soil layer.

Water can dissolve phosphorus as a solvent under suitable soil moisture concentration, which can reduce total phosphorus and increase available phosphorus (Williams et al., 2018). However, this process of change is a relatively slow process, so the change is small. The abovementioned phosphorus accumulation and total phosphorus uptake in plants first increased and then decreased in 2017 and 2018 and reached a maximum in the P_2_ treatment, which was significantly greater than that in the P_3_ treatment (Table 4). However, the total phosphorus in the P_3_ treatment was greater than that in the P_2_ treatment in the 0–20 cm soil layer (Figure 3). The phosphorus application in the P_3_ treatment was higher than that in the P_2_ treatment and was not better absorbed by plants, leading to further accumulation of total p and available p in the soil. Therefore, too much phosphorus application leads to low alfalfa yield, soil environmental pollution, and economic waste. In this study, total phosphorus and available phosphorus in the phosphorus application treatments decreased gradually over the years compared with that in the P_0_ treatment in the 0–20 cm soil layer (Figures 3 and 4). It could be concluded that alfalfa cultivation on the gray desert soil would reduce the original available phosphorus concentration, deplete the soil nutrients, reduce soil fertility, and reduce the yield at the same time.

In summary, when the phosphorus amounts in soil are lower than that required by plants, the consumption of phosphorus in soil is greater than that accumulated, and the phosphorus nutrient level in soil decreases gradually; when the amount of phosphorus in soil is higher than that required by plants, the phosphorus pool in the soil will increase continuously. Therefore, to maintain the balance of soil phosphorus accumulation and consumption and to save phosphorus resources in agricultural production, the minimum required amount of phosphorus should be the amount of fertilizer used to maintain the balance of soil phosphorus input and consumption. In this way, the economic risks of fertilization and the environmental pollution of the soil are avoided while the profit per unit area from fertilization is guaranteed.

### 4.4 Membership function analysis for evaluation of the optimum combination of phosphorus application and irrigation

In this study, the accumulated phosphorus concentration was significantly positively correlated with total phosphorus and available phosphorus concentration (*p* < .01). Total phosphorus was significantly positively correlated with available phosphorus (*p* < .01) (Table 6). Available phosphorus is usually used to measure the phosphorus nutrient status of soil in long‐term agricultural production, which indicates that the available phosphorus concentration is limited by the supply of total phosphorus in soil. Maintaining the soil total phosphorus concentration is beneficial for increasing the available phosphorus level, thus ensuring the supply of phosphorus nutrients for alfalfa. The effects of different phosphorus application and irrigation amounts on the hay yield, accumulated phosphorus concentration, total phosphorus uptake, water‐use efficiency and phosphorus‐use efficiency of alfalfa, and soil available phosphorus and total phosphorus were different. The evaluation of the optimal model for water and phosphorus through these indicators did not fully clarify the advantages and disadvantages of the different treatments, and the membership function analysis method could be used to evaluate multiple optimal indicators by synthesizing multiple indicators. In this study, it was found that the optimal water–phosphorus management mode was the W_2_P_2_ treatment, that is, irrigation at 6,000 m^3^/ha and P application at 100 kg/ha (Table 7). This was an appropriate water–phosphorus combination model for high quality and high yield alfalfa production under drip irrigation. This treatment effectively improved the hay yield of alfalfa, promoted the absorption of available phosphorus by alfalfa plants, improved the water‐use efficiency and phosphorus‐use efficiency, increased the available phosphorus concentration in soil, and reduced the total phosphorus concentration.

## 5 CONCLUSION

Moderate management of water and phosphorus was the key measure for high efficiency alfalfa production under drip irrigation. Phosphorus application significantly improved the water‐use efficiency and played a role in water transfer. During the whole growing season of alfalfa, the phosphorus‐use efficiency was higher under the low fertilizer levels, but the effect of phosphorus application on the water‐use efficiency was not obvious. Phosphorus application significantly increased the available phosphorus concentration in the 0–20 cm soil layer, and available phosphorus gradually decreased with increasing soil depth. The optimal water–phosphorus combination was irrigation at 6,000 m^3^/ha and phosphorus application at 100 kg/ha per year, which resulted in high quality and high yield of alfalfa under drip irrigation.

## CONFLICT OF INTEREST

The authors declare that they have no conflict of interest.

## ETHICAL APPROVAL

The study did not involve any human or animal testing.

## INFORMED CONSENT

Written informed consent was obtained from all study participants.
